# Diffusion imaging of whole, *post-mortem* human brains on a clinical MRI scanner

**DOI:** 10.1016/j.neuroimage.2011.03.070

**Published:** 2011-07-01

**Authors:** Karla L. Miller, Charlotte J. Stagg, Gwenaëlle Douaud, Saad Jbabdi, Stephen M. Smith, Timothy E.J. Behrens, Mark Jenkinson, Steven A. Chance, Margaret M. Esiri, Natalie L. Voets, Ned Jenkinson, Tipu Z. Aziz, Martin R. Turner, Heidi Johansen-Berg, Jennifer A. McNab

**Affiliations:** a FMRIB Centre, Nuffield Department of Clinical Neurosciences, University of Oxford, Oxford, UK; b Clinical Neurology, Nuffield Department of Clinical Neurosciences, University of Oxford, Oxford, UK; c Nuffield Department of Surgical Sciences, University of Oxford, Oxford, UK; d A.A.Martinos Centre, Massachusetts General Hospital, Boston, USA

**Keywords:** Diffusion tensor imaging, Tractography, *Post mortem*, Human, Brain

## Abstract

Diffusion imaging of *post mortem* brains has great potential both as a reference for brain specimens that undergo sectioning, and as a link between *in vivo* diffusion studies and “gold standard” histology/dissection. While there is a relatively mature literature on *post mortem* diffusion imaging of animals, human brains have proven more challenging due to their incompatibility with high-performance scanners. This study presents a method for *post mortem* diffusion imaging of whole, human brains using a clinical 3-Tesla scanner with a 3D segmented EPI spin-echo sequence. Results in eleven brains at 0.94 × 0.94 × 0.94 mm resolution are presented, and in a single brain at 0.73 × 0.73 × 0.73 mm resolution. Region-of-interest analysis of diffusion tensor parameters indicate that these properties are altered compared to in *vivo* (reduced diffusivity and anisotropy), with significant dependence on *post mortem* interval (time from death to fixation). Despite these alterations, diffusion tractography of several major tracts is successfully demonstrated at both resolutions. We also report novel findings of cortical anisotropy and partial volume effects.

## Research highlights

► Acquisition and processing protocols for diffusion MRI of *post-mortem* human brains. ► Effect of *post-mortem* and scan intervals on diffusion indices. ► Tractography in *post-mortem* human brains. ► Radial diffusion anisotropy in cortical gray matter.

## Introduction

Diffusion-weighted MRI has become a popular method for investigating white matter non-invasively. It has great potential for probing both white matter microstructure, using indices such as fractional anisotropy (FA), and macrostructure, based on tracing of fiber tracts (“tractography”). Although there is now substantial literature reporting the use of diffusion imaging across a broad range of white matter regions, species, and pathologies, the link between this data and the (even richer) literature based on classical examination of *post mortem* tissue (dissection or histological staining) is relatively sparse.

A number of studies have demonstrated the feasibility and utility of diffusion imaging of *ex vivo* animal brains (Guilfoyle et al., 2003; Verma et al., 2005; Kroenke et al., 2005; D'Arceuil et al., 2007, 2008; Dyrby et al., 2007; Tyszka and Frank, 2009), spinal cord (Schwartz et al., 2005; Kim et al., 2009) and brain tissue sections (Guilfoyle et al., 2003; D'Arceuil et al., 2005). These studies have utilized small-bore, high-field scanners, typically with a maximum gradient amplitude of 400 mT/m or greater (10 times that available on most clinical systems). These specialized systems are ideal for *ex vivo* scanning because they are able to achieve high b-values (indicating strong diffusion contrast) with short echo times (enabling high signal-to-noise ratio, SNR). Unfortunately these systems typically have a bore size that is too small to fit whole human brains, and are less commonly available than human scanners, particularly in a clinical setting.

Although much can be learned from these studies on animal brains and spinal cord, the possibility of scanning whole human brains is particularly compelling. The use of human tissue is critical to study uniquely-human pathologies where animal models are inappropriate or limited, such as psychiatric disorders, high-level cognitive dysfunction or even multiple sclerosis. Moreover, the validation of long-range tracts in human brains is important, and would be particularly valuable in the context of conditions affecting global connectivity, such as schizophrenia and autism. This data could also go beyond what is achievable *in vivo*, enabling higher spatial resolution. Routine scanning of whole human brains donated to brain bank facilities could be used to provide databases of matched diffusion and histology, provided MRI scans could be obtained reliably and at a reasonable expense. Given that most brain bank resources are sited in or near research hospitals, this could be achieved provided clinical scanners could provide sufficiently good data. In the present work, we consider the longest conceivable scan time in a hospital setting, 24 h. Ultimately we would hope to reduce this time to an overnight scan.

Several studies have previously acquired diffusion-weighted data in whole, *post mortem* human brains (Pfefferbaum et al., 2004; Larsson et al., 2004) or brain slices (Schmierer et al., 2007; Gouw et al., 2008). Unfortunately, changes in tissue properties with fixation compromise conventional sequences. Large voxel dimensions are typically prescribed to combat reductions in SNR due to shortened T_ 2_. In addition, the reductions in diffusion coefficient are rarely compensated for with increased b-value, resulting in lower overall sensitivity to diffusion. Finally, the use of single-shot EPI introduces a tradeoff between image resolution and distortion. As a result, the image quality and diffusion contrast in these studies are generally worse than those achievable *in vivo*. These issues make straightforward application of protocols developed for *in vivo* imaging inappropriate for many of the goals discussed above.

In this manuscript, we present initial results demonstrating the feasibility of scanning whole, fixed, human brains on a clinical 3 T scanner. The approach considered here can be achieved with straightforward modification of conventional spin-echo diffusion sequences. Instead of acquiring data using a single-shot EPI readout, as is used *in vivo*, we acquire data using a 3D, segmented EPI acquisition. We explore the achievable data quality when scan time is limited to a 24-hour period, and also present data at higher resolution from a 5-day scan. We study the impact of tissue preparation on the derived diffusion indices, present tractography results from major pathways and discuss some interesting properties of our diffusion data.

## Background

### Previous findings in fixed tissue

Death and fixation causes a number of changes to tissue properties that affect MR imaging. Fixed tissues suffer reduced proton density and T_ 2_(Pfefferbaum et al., 2004), generally increasing the number of averages required to achieve reasonable SNR. In addition, the diffusion coefficient is drastically reduced (Sun et al., 2003, 2005; D'Arceuil et al., 2007), requiring higher b-values in order to obtain comparable diffusion contrast to *in vivo* experiments. A number of studies have considered whether FA is preserved in fixed white matter, with early work finding it unchanged (Guilfoyle et al., 2003; Sun et al., 2003, 2005; D'Arceuil et al., 2007), but several more recent papers suggesting that it may be reduced (Madi et al., 2005; Schmierer et al., 2007; D'Arceuil and de Crespigny, 2007). These studies varied in several potentially important details of fixation, including the fixation method (perfusion versus immersion fixation), *post mortem* interval (PMI, time from death to fixation) and scan interval (SI, time from death to scan). At least one study has demonstrated the importance of PMI on FA and ADC (D'Arceuil and de Crespigny, 2007), while another has suggested that tissue is stable for SI of up to 3 years (Dyrby et al., 2011).

### Proposed approach

The reduced T_ 2_, proton density and diffusion coefficient in fixed tissue are generally unfavorable for diffusion-weighted imaging; however, there is potential for more flexibility in acquisition strategy than exists *in vivo*. The lack of motion in pom scans means that the single-shot acquisitions generally used for *in vivo* diffusion imaging are not necessary, enabling the use of acquisition techniques that would suffer from severe image artifacts *in vivo*. Previous work has taken advantage of this flexibility by using line-scan (Guilfoyle et al., 2003; D'Arceuil et al., 2007; Dyrby et al., 2011), segmented EPI (Englund et al., 2004), 3D echo trains (Tyszka and Frank, 2009) and 3D segmented EPI trajectories (D'Arceuil and de Crespigny, 2007). The latter method has the attractive property of being highly efficient while being achievable through modification of the spin-echo EPI sequences used for *in vivo* diffusion imaging. This method has been our primary technique for imaging whole, human brains.

Spin-echo imaging does, however, suffer from a tradeoff between SNR (favoring short T_E_ to minimize T_ 2_ signal loss) and contrast (with large b-value requiring long T_E_). This tradeoff is particularly problematic for the reduced T_ 2_ and ADC of fixed (compared to *in vivo*) tissue. In other work, we explore the possibility of *post mortem* diffusion imaging with a steady-state free precession sequence (McNab et al., 2009a), which has potential to overcome this problem.

## Methods

### Subject pathology

The brains reported in this study represent a broad range of specimen types. These include one brain with no known pathology (CTL01, cause of death unknown), one diagnosed with autism spectrum disorder (ASD02), one diagnosed with motor neurone disease (MND01) and eight diagnosed with multiple sclerosis (MSxxx). Brains were extracted from the cranium and immersion fixed in a 10% neutral buffered formalin. The PMI was 46.2 ± 19.9 (21–69) h and the SI was 25.2 ± 14.5 (2–40) months. These brains were all scanned with the same imaging protocol.

Finally, one brain was scanned at higher resolution than the others. This patient had a fairly complicated history (described previously in McNab et al., 2009b) including bipolar disorder and a right thalamic stroke. This subject had an *in vivo* diffusion scan as part of pre-surgical planning that is also presented here for comparison.

### Scanning and tissue preparation

All imaging was performed on a Siemens Trio 3 T scanner using a 12-channel head coil for signal reception. All scans were performed in a single session without removing the brain from the scanner, even for the high-resolution, 5-day experiment.

For imaging, brains are transferred to a close-fitting plastic container. Some previous studies have scanned brains in fixative solution (Englund et al., 2004; Kroenke et al., 2005). However, we found this to introduce image degradation due to intense signal (which limits dynamic range and introduces ringing artifacts), the need for larger field-of-view and chemical shift. To avoid these problems, we immerse each brain in a proton-free fluid, Fomblin LC/8 (Solvay Solexis Inc.), which has no MR signal and is susceptibility matched to tissue (Benveniste et al., 2000; D'Arceuil et al., 2007; D'Arceuil and de Crespigny, 2007). Although we do not report matched MRI-histology results in this work, we do note that residual Fomblin was found to necessitate hand (rather than automated) embedding for subsequent histological staining. To date, no damage to tissue exposed to Fomblin has been observed.

Brains are transferred into the imaging container 12–24 h prior to scanning to allow air bubbles to escape and the brain to warm to room temperature. This relatively simple approach yielded reasonable image quality without significant degradation due to air bubbles, although other groups have actively eliminated bubbles with vacuum pumping (D'Arceuil et al., 2007). Another approach is to set the brain in agar gel (Pfefferbaum et al., 2004), although great care must be taken to avoid bubbles. Embedded brains are also prone to conduct table vibration into the embedding gel caused by imaging gradients. All scanning was performed at room temperature (approximately 20 °C).

Based on previous work, we explored the possibility of soaking the brain in a Gadolinium-doped buffer solution prior to imaging (D'Arceuil et al., 2007). Previous work suggests that the buffer increases the tissue T_ 2_ (D'Arceuil et al., 2007), apparently by replacing fixative (short T_ 2_) with buffer (long T_ 2_) (Shepherd et al., 2009). Gadolinium doping reduces the tissue T_ 1_, thereby reducing the T_ 1_ saturation effects at short T_R_ (D'Arceuil et al., 2007). As discussed below, we found this pre-soaking to be problematic in human brains (see Supplementary Fig. 1), and have abandoned this procedure.

### Diffusion protocols

**Fig. 1:**
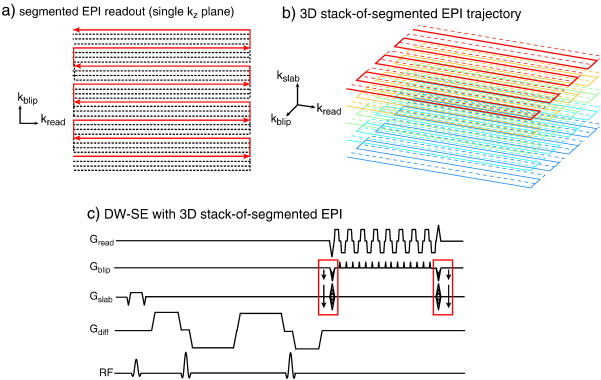
Modified spin-echo diffusion sequence used in this study. The vendor product sequence was modified to transform the readout from single-shot EPI into a 3D, stack-of-segmented EPI trajectory. (a) Each 2D k-space plane is acquired in a series of segments, covering interlacing sets of k-space lines (one segment is shown as the red solid line). (b) These segmented 2D planes (color coded here) are stacked to fill out the third k-space dimension. (c) The sequence uses a standard twice-refocused diffusion weighting scheme, followed by the 3D EPI readout. Modified readout gradients are indicated by the red boxes.

The diffusion-weighted sequences implemented in this work use 3D, segmented-EPI acquisitions, illustrated in Fig. 1. Diffusion-weighted spin echo (DW-SE) imaging was implemented with fairly minor modifications to the 2D single-shot EPI sequence used *in vivo*. In particular, this sequence uses a twice-refocused diffusion-weighting scheme, which reduces eddy-current-induced image distortions (Reese et al., 2003). The use of a 3D readout, which excites the entire imaging volume each T_R_, makes the sequence compatible with relatively short T_R_ and improves SNR efficiency. The optimum T_R_ is dependent on the tradeoff between T_ 1_ recovery (favoring longer T_R_) and the SNR advantage of acquiring signal as often as possible (favoring shorter T_R_). For fixed white matter (T_ 1_≈ 340 ms; McNab et al., 2009a), the optimal T_R_ is in the range of 500–700 ms. This short T_R_ would be incompatible with 2D, multi-slice readouts used in conventional diffusion imaging.

Our primary protocol achieves 0.94 × 0.94 × 0.94 mm resolution with diffusion weighting of b = 4500 s/mm^2^. Imaging parameters for this protocol include: T_E_ / T_R_ = 122/530 ms, flip angle 75°, bandwidth 789 Hz/pixel, 32 lines per EPI segment, matrix size 168 × 192 × 120, partial Fourier factor 5/8 along the blip direction, and acquisition time per volume of 6:22. In total, images were acquired with 54 isotropically-distributed diffusion-encoding directions and 6 b = 0 images were acquired for each repetition of the protocol. The entire protocol was repeated 3 times. We present data from eleven *post mortem* brains scanned with this protocol.

A single experiment in one brain was performed to explore the feasibility of higher spatial resolution. This experiment achieved a voxel size of 0.73 × 0.73 × 0.73 mm with b = 3050 s/mm^2^. Although the linear voxel dimensions are only marginally smaller than for the 24-hour protocol, the voxel volume is more than twice as small, leading to a need to scan at least four times longer. We acquired this data over approximately 5 days of continuous scanning (4 days of diffusion scanning). Imaging parameters for this protocol include: T_E_ / T_R_ = 114/670 ms, flip angle 77°, bandwidth 820 Hz/pixel, 32 lines per EPI segment, matrix size 254 × 254 × 192, partial Fourier factor 5/8 along the blip direction and acquisition time per volume of 17 min. 64 isotropically-distributed diffusion encoding directions and 5 b = 0 images were acquired for each average, and the entire protocol was repeated 5 times. The total acquisition time for diffusion data was just under 100 h. An *in vivo* scan had been acquired in this patient several years before death as part of pre-surgical planning. *In vivo* acquisition parameters included: 1.5 T scanner, voxel size 2 × 2 × 2 mm, T_E_ / T_R_ = 97/10,100 ms, flip angle 90°, bandwidth 1860 Hz/pixel, matrix size 128 × 104 × 64, partial Fourier factor 6/8. In total, this protocol acquired 3 repeats of 60 isotropically-distributed diffusion-encoding directions with b = 1000 s/mm^2^, plus 27 repeats with b = 0 s/mm^2^.

### Structural protocols

Structural scans were acquired in the same session to ensure good alignment and similar tissue deformation to diffusion scans (the slight deformations introduced by packing the brains will in general change if the brain is removed from the container between scan sessions). T_1_-weighted structural protocols similar to those used *in vivo* for obtaining good gray–white contrast exhibited poor contrast in *post mortem* samples. This effect has been observed before and is attributed to the convergence of e values in fixed gray and white matter (Pfefferbaum et al., 2004), which we have previously measured at 300 and 340 ms, respectively (McNab et al., 2009a). To improve gray–white contrast, we instead use a 3D balanced steady state free precession (BSSFP) pulse sequence using T_E_ / T_R_ = 3.7/7.4 ms and flip angle 35°. This protocol results in high-contrast scans with higher signal in gray matter than white matter (the opposite pattern to *in vivo* T_ 1_ structurals). BSSFP images are acquired in pairs with the RF phase incrementing 0° and 180°, which are averaged to reduce banding artifacts (Vasanawala et al., 2000). Our standard structural protocol is acquired at 0.5 × 0.5 × 0.5 mm resolution (bandwidth 302 Hz/pixel, matrix 352 × 330 × 416, 16 min per pair). This protocol is repeated for 1–2 h to increase SNR. The higher-resolution diffusion scan was accompanied by a structural at 0.33 × 0.33 × 0.33 mm resolution (bandwidth 395 Hz/pixel, matrix 576 × 576 × 480, 32 min per pair).

### Data pre-processing

**Fig. 2:**
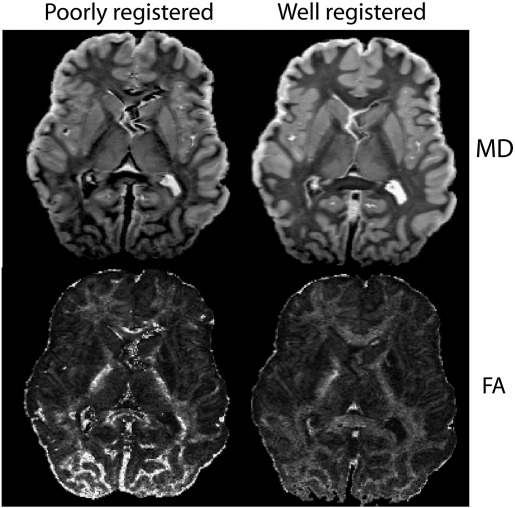
Effect of registration fidelity on final diffusion parameter maps (MD and FA). Even with the twice-refocused diffusion preparation, eddy currents introduce image distortions (primarily scaling and shearing) that vary with diffusion direction. The processing pipeline traditionally used in our laboratory, which simply applies 12-degree-of-freedom alignment to the raw diffusion-weighted images, does a poor job of aligning the data. This leads to artifactual areas of low MD and high FA. Our modified processing pipeline is able to estimate eddy-current effects more effectively and significantly reduces these artifacts.

Individual diffusion scans are co-registered using FLIRT (Jenkinson and Smith, 2001) to correct for B_ 0_ drift and eddy-current distortions. Although the basic steps are the same as *in vivo*, the very low signal levels in individual diffusion-weighted images necessitated alterations to certain aspects of the pre-processing pipeline. The first stage is to remove B_ 0_-induced image drift (caused by gradient-induced heating of the scanner) from each repeat of the protocol using registration constrained to remove shifts along the slowest phase-encode direction. Repeats of each diffusion direction are then averaged, at which point eddy-current motion (consistent across repeats) is the dominant source of misregistration between different diffusion directions. These effects are removed with affine registration including shear and scale terms (12 degrees of freedom). This correction is critically dependent on the use of a mutual-information-based cost function. These relatively minor alterations to the pre-processing pipeline have a major affect on the quality of subsequent analysis, as demonstrated in Fig. 2. The registration of the b = 0 scans to the structural scan is more straightforward. In particular, the quality of this alignment is excellent due to the low levels of distortion in the multi-shot diffusion data, such that no distortion unwarping is necessary.

### Tensor analysis

FMRIB's Diffusion Toolbox (FDT), part of the FMRIB Software Library (FSL) (Smith et al., 2004), was used to fit a diffusion tensor model to the data at each voxel. Maps of FA, MD, radial and axial diffusivity (D_rad_ and D_ax_), and principal diffusion direction are generated by this analysis. Region-of-interest (ROI) masks were hand-drawn for five white matter and three gray matter regions: corpus callosum (CC), superior longitudinal fasciculus (SLF), cingulum bundle (Cing), optic radiations (Opt), posterior limb of the internal capsule (PLIC), thalamus (Thal), caudate (Caud) and putamen (Put). Care was taken to avoid any obvious lesions, which were present in some of the brains. Example masks for subject CTL01 are given in the Supplementary Material. The mean FA, MD, D_rad_ and D_ax_ were extracted from the ROIs. Regression of the diffusion indices against PMI and SI was performed to test for effects relating to the delay in fixation and duration of fixation.

### Tractography analysis

BEDPOSTX was used for Bayesian estimation of a two-fiber model (*i.e.*, up to two-fibers plus an isotropic compartment) using Markov chain-Monte Carlo (MCMC) sampling (Behrens et al., 2007). This provides a voxel-wise estimate of the angular distribution of local tract direction for each fiber, which is the starting point for tractography. MCMC sampling was found to require a longer burn-in period for the 0.73 mm data (5000 iterations) to ensure proper convergence, while the 0.94 mm data converged with the standard parameters (1000 iterations).

BEDPOSTX usually uses automatic relevance determination (ARD) to determine whether the data contains strong evidence for a second fiber, and if not, uses a simpler one-fiber model. For some known tracts (here, the corticospinal tract), accurate tractography was only possible when the ARD was replaced with a uniform prior, which forces estimation of the second fiber population in every voxel. (This is accomplished in BEDPOSTX by setting the ARD parameter to 0.)

Tractography was then performed from a series of hand-drawn seed masks using the Probtrackx probabilistic tractography software (Behrens et al., 2007). Probtrackx repetitively samples from the voxel-wise posterior distribution of fiber orientations, each time computing a streamline through the local samples. The fiber tracts reconstructed were chosen to represent the main categories of fiber types and tract directions: the corticospinal tract (CST, projection fibers), cingulum and fornix (association fibers) and corpus callosum (CC, commissural fibers).Tractography masks were drawn on the structural scans. Two strategies for tractography were considered. The more straightforward strategy defined a small seed mask in the tract of interest and was used for tractography of the high-resolution data. The second method is a “global” approach: streamlines are seeded over a large 3D FOV that encapsulates the tract of interest. Inclusion masks used to define two regions that each tract must pass through, and only streamlines that pass through both regions are retained. This method was used for the lower-resolution data.

### Histology

In order to assist in our interpretation of diffusion properties at the gray–white boundary, histological images were obtained from a separate brain specimen from a subject with no known pathology. Five-mm blocks were cut from the temporal lobe orthogonal to its long axis. Blocks were paraffin embedded and sectioned to provide 25 micron thick sections. Sections were stained using standard protocols for Luxol Fast Blue to stain myelin and counter-stained with Cresyl Violet to visualize Nissl substance in cell bodies. Digital photomicrographs were captured with a digital camera attached to an Olympus BX40 microscope using 4× and 60× objective lenses.

## Results and discussion

### Structural scans

**Fig. 3:**
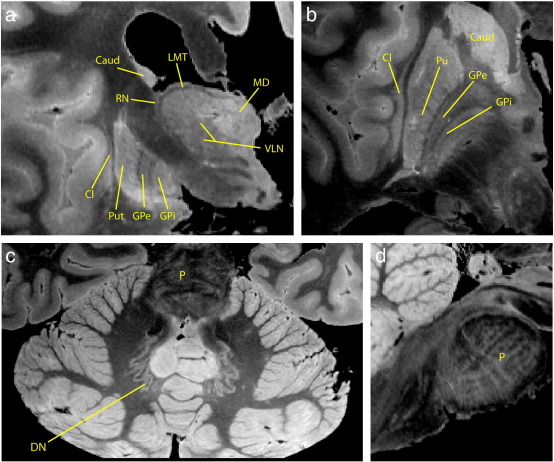
Example structural images acquired with phase-cycled SSFP in a *post mortem* human brain at 0.33 mm resolution. Coronal (a) and axial (b) slices through the thalamus, globus pallidus, and white matter capsules demonstrating clear delineation of small substructures. GPe = external globus pallidus, GPi = internal globus pallidus, Cl = claustrum, Put = putamen, Caud = caudate, MD = dorso-medial thalamic nucleus, VLN = ventrolateral thalamic nucleus, RN = reticular nucleus, LMT = medullary thalamic lamina. (c) Axial slice through the cerebellum and pons depicting cerebellar structures including the pontine fibers (P) and dentate nucleus (DN). (d) Sagittal slice through the brainstem showing decussating pontine fibers (P).

A representative structural scan is shown in Fig. 3 with labels identifying a number of deep brain structures. The small voxel size and high SNR of these images enable visualization of a number of structures that are difficult to distinguish in T_ 1_-weighted structurals *in vivo*. Some structures, such as the external and extreme capsules and the internal and external globus pallidus, can be differentiated because the thin structures separating them are resolved. Similarly, the dentate nucleus and pontine fibers, which are rarely visible *in vivo* due to their small size, are conspicuous in these images. Larger structures, such as the dorso-medial and ventro-lateral nuclei in the thalamus, benefit from the high contrast-to-noise ratio. In addition to their intrinsic value as an anatomical reference, these high-quality structurals, combined with the well-matched distortion in the diffusion data, enable greatly improved accuracy for seeding diffusion tractography, such as the external/extreme capsule results presented below.

Previous studies in animal brains soaked the tissue before imaging it in either a buffer solution (aimed at increasing the tissue T_ 2_) or a buffered gadolinium solution (reducing T_ 1_, which enables shorter T_R_ and improves imaging efficiency). Several of our brains were pre-soaked for 48 h prior to scanning, which in conjunction with the 24-hour scan was deemed to be the maximum duration over which specimens could be out of fixative without tissue degradation. However, this pre-soaking was detrimental to image quality, primarily because the buffer was not able to penetrate to the interior of the brain in the allotted time, resulting in a buffer-induced contrast boundary that severely obscures the underlying tissue contrast (Supplementary Fig. 1). The buffer soaking has been abandoned and was not used in the data presented here.

### 24-hour results

**Fig. 4:**
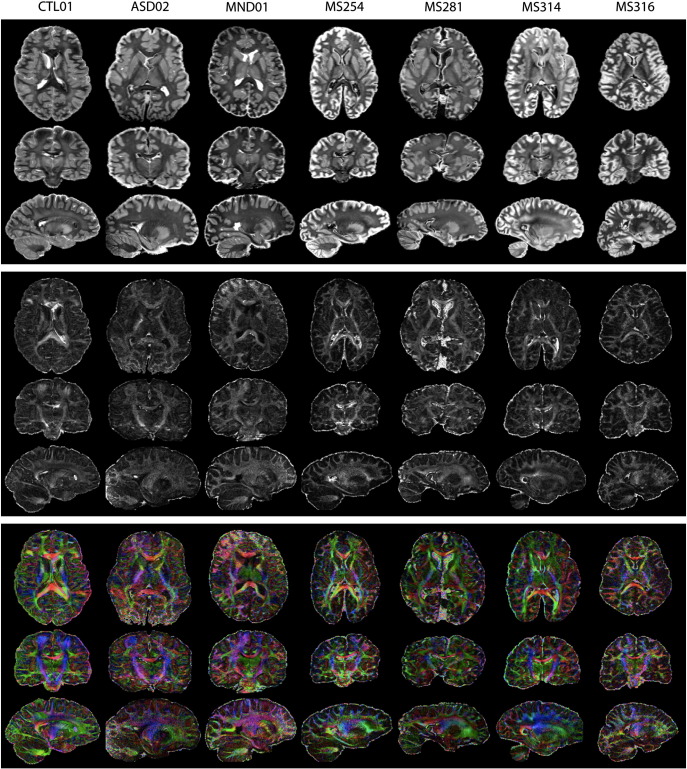
Diffusion indices derived from the first seven brains scanned using the 24-hour DW-SE protocol at 0.94 mm resolution. The indices displayed are MD (top), FA (middle) and the principal diffusion direction (PDD, bottom, color-coded and weighted by FA).

Images for the first seven brains scanned with the 24-hour DW-SE protocol are shown in Fig. 4. Image quality is overall quite good, but variable across brain specimens. Several scans have a noticeable drop in SNR over part of the brain (*e.g.*, the anterior pole of MND01 and posterior pole of ASD02), which appears to be due to inappropriate placement of the brain within the receive array coil. Although this does not affect the mean diffusion indices calculated in these regions (since the data is referenced to b = 0 scans with the same effect), it does result in high variance in these regions and makes alignment more difficult. Several brains exhibit residual protonated fluid in the ventricles where the proton-free fluid had not replaced fixative (characterized by high signal at b = 0 and no signal high b-value, leading to high MD and low FA). This signal is a slight nuisance due to its variability, but is not particularly problematic for analysis. Despite variable SNR, we find very consistent tensor fit across all brains.

**Fig. 5:**
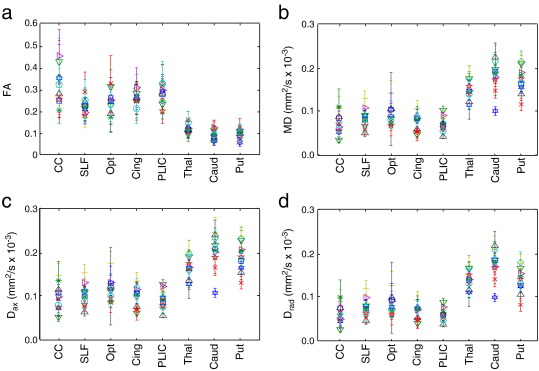
(a–d) Diffusion indices calculated from eight hand-drawn ROIs in eleven brains (the same data used in the PMI and SI regression analyses given below).

The contrast observed in MD and FA images differs considerably in our data compared to *in vivo* diffusion data, with strong gray–white contrast in the MD images and relatively weak contrast in the FA maps (whereas *in vivo* images exhibit little gray–white contrast in MD and strong contrast in FA). The principal eigenvector of the tensor fit is clearly defined in all brains and has good directional correspondence to *in vivo* data. Tensor-derived parameters in a range of white and gray matter ROIs are given in Fig. 5 and Table 1 (these values in individual specimens are provided in the Supplementary Material). As can be seen in the tensor maps, the *in vivo* relationship of higher FA in white matter compared to gray matter is roughly preserved *post mortem* (the mean FA being significantly different between gray and white with p < 0.05). Similarly, the CC is observed to have highest FA of all white matter regions, similar to *in vivo* data.

Nevertheless, the diffusion properties of tissue do differ significantly compared to *in vivo* brain tissue. Most prominently, MD and FA values are considerably lower than found *in vivo*, by factors of approximately 10 and 2–3, respectively. Changes in MD have been reported previously and attributed to the combined effects of death, fixation and reduced temperature (Schwartz et al., 2005; Kim et al., 2007; D'Arceuil and de Crespigny, 2007; Widjaja et al., 2009); however, animal studies have generally only reported reductions to 25–30% of *in vivo* values (Sun et al., 2003, 2005; D'Arceuil et al., 2007). Our data also exhibit strongly reduced FA values in white matter compared to *in vivo*. Although several animal studies have reported preserved FA (Guilfoyle et al., 2003; Sun et al., 2003, 2005; D'Arceuil et al., 2007), the reduced FA values in white matter have some precedence in the literature (Madi et al., 2005; Schmierer et al., 2007; D'Arceuil and de Crespigny, 2007).

**Fig. 6:**
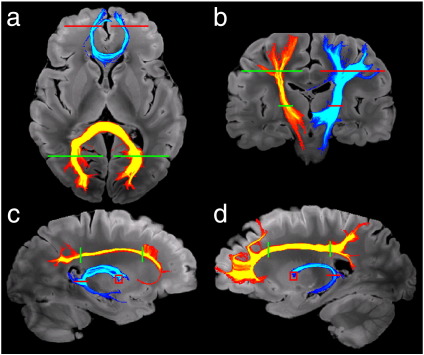
Tractography performed on brain CTL01 demonstrating a range of major white matter tracts. The inclusion masks for each tract are indicated by the green or red lines. (a) Corpus callosal tracts passing through the genu (blue) and splenium (yellow). (b) Right and left corticospinal tracts. (c and d) Cingulum (yellow) and fornix (blue) from the left and right hemispheres. Tracts are displayed as maximum-intensity-projections on top of the structural scan.

Tractography results for CTL01 are shown in Fig. 6. These tracts were generated using the “global” approach described above, where tracts were seeded from a large 3D region encapsulating the tract (in principle, seeding would be done from the entire brain, but was restricted here to reduce computation time). All streamlines that pass through both of the inclusion masks are considered part of the tract of interest and included in the final probability map. The inclusion masks are roughly indicated in Fig. 6; no exclusion masks were used. Five major tracts with known anatomy were tracked in this brain. One commissural tract, the corpus callosum, was traced in two regions: the forceps minor, corresponding to the CC genu, and the forceps major, corresponding to the CC splenium. Two association fiber tracts were traced: the cingulum and the fornix, both in the right and left hemispheres. These tracts used standard BEDPOSTX output, generated using automated detection of the number of fibers (in BEDPOSTX, setting ARD = 1). The final tracts, the right and left CST, had high uncertainty on the second fiber direction and could only be traced when estimation of the second fiber population was enforced in all voxels (ARD = 0). Without this, streamlines diverged in the region where the CST crosses the SLF and the callosal radiations, effectively terminating the tracts. In these regions, the tensor fits have low FA and appear to have a dominant right–left direction, rather than superior–inferior.

### 5-day results

**Fig. 7:**
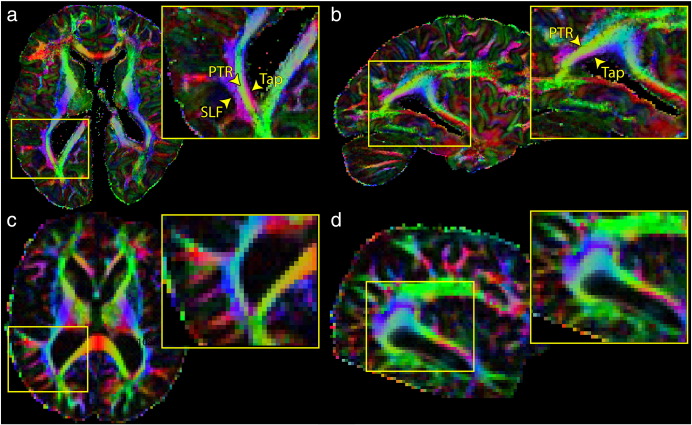
Post-mortem (0.73 × 0.73 × 0.73 mm^3^) and *in vivo* (2 × 2 × 2 mm^3^) diffusion data in the same patient. (a and b) High-resolution data disambiguates a number of tracts that often cannot be differentiated *in vivo*. For example, the tapetum (Tap) of the corpus callosum is clearly differentiated from the posterior thalamic radiation (PTR) and the superior longitudinal fasciculus (SLF). (c and d) The PTR and SLF are much less conspicuous in the *in vivo* data, and the tapetum cannot be distinguished from the PTR (in this or any slice). Note that some of the improvement in data quality may also be due to the complete lack of motion in the *in vivo* data.

Fig. 7 shows color maps of the principal eigenvector in the high-resolution (0.73 mm) data, with the equivalent slices from the same subject *in vivo* for comparison. The resolution of the *post-mortem* scan clearly delineates structures that cannot be distinguished *in vivo*. For example, near the posterior horn of the lateral ventricles, the superior longitudinal fasciculus, posterior thalamic radiations and tapetum are clearly disambiguated. These structures are at best a few voxels thick *in vivo*, and the tapetum is completely concealed by partial volume effects.

**Fig. 8:**
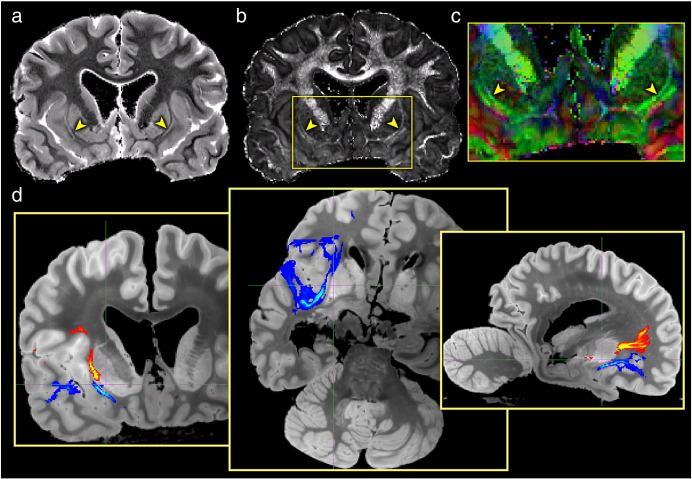
(a and b) High-resolution maps of MD and FA (respectively) in the coronal orientation. These maps show a clear distinction between the external and extreme capsules (arrowheads). (c) A zoomed view of the color representation of the principal diffusion direction shows that the fibers through the external and extreme capsules run parallel to each other in this region, separated only by the thin gray matter of the claustrum (dark line near arrowheads). Note that this slice is taken through the anterior limb of the internal capsule, where the fibers have a significant anterior–posterior orientation (the large green tracts at the top of the image). (d) Seeding separately from the external and extreme capsules probabilistic tractography distinguished the inferior fronto-occipital fasciculus (shown in red-yellow) which runs mainly through the external capsule in the superior regions and the uncinate fasciculus (shown in blue) which runs mainly through the extreme capsule in the more inferior regions.

In the coronal view, we can also see a division between the external and extreme capsules (Figs. 8a–c, arrows). These tracts are separated by a thin layer of gray matter, the claustrum, and form an important white matter bridge between the temporal, occipital and frontal lobes. Disambiguation of these tracts has been noted to be beyond the limits of current *in vivo* imaging (Mori et al., 2005). We tracked the external and extreme capsules using a conventional seed-based approach (as contrasted with the global approach taken above). The resulting tracts distinguish the inferior occipito-frontal fasciculus which runs mainly through the external capsule in the superior regions and the uncinate fasciculus which runs mainly through the extreme capsule in the more inferior regions (Kier et al., 2004), as shown in Fig. 8d. At conventional resolution, these tracts often bleed together due to their proximity.

A second example of high-resolution tractography is the stria terminalis and the fornix, two thin white matter tracts that also run closely together. We were able to reconstruct the posterior portion of these tracts with the stria terminalis extending from the amygdala and the fornix extending from the hippocampus. These results are available in the Supplementary Material (Fig. 4). We have previously traced these tracts using high-resolution, *post mortem* DW-SSFP data (McNab et al., 2009a).

### Diffusion properties: effect of PMI and SI

Above, we note that the diffusion indices measured with our 24-hour protocols depart significantly from *in vivo* measurements. Although we did not conduct specific experiments aimed at elucidating the source of these differences, we can do some limited analyses and literature comparisons to identify the most likely causes.

One significant difference between our study and the prior literature on *post mortem* diffusion scanning (mostly based on animal tissue) is the long and variable delays from death to fixation (*post-mortem* interval, PMI) and death to scan (scan interval, SI). PMI has previously been implicated for changes to MD and FA in a study specifically aimed at predicting these effects in human cadavers stored in morgue conditions (D'Arceuil and de Crespigny, 2007). To test for parameter dependence on tissue preparation, we regressed diffusion indices against PMI and SI. Scatter plots including single regressions are shown for ease of illustration, while multiple regressions are used to assess statistical significance (this analysis provides a linear adjustment for the correlation r = 0.32 between PMI and SI in our specimens).

**Fig. 9:**
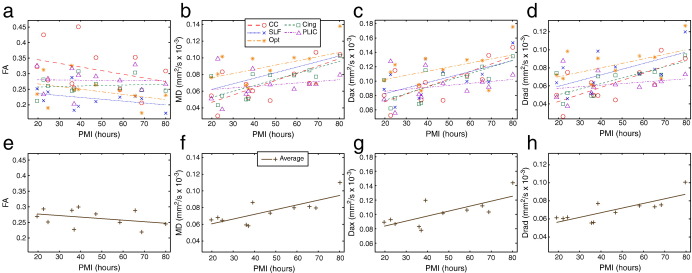
(a–d) Single regressions of tensor-derived parameters against PMI for five white-matter regions (CC, SLF, Opt, Cing and PLIC). For any given tensor parameter, at least one region exhibited a statistically-significant dependence on PMI. However, the diffusivity parameters exhibited a stronger and more significant dependence overall than FA. (e–h) Single regressions of the same tensor parameters against the average across the five white matter regions.

Results of single regression of FA, MD, D_ax_ and D_rad_ from five white matter regions onto PMI are depicted in the top row of Fig. 9. A strong dependence of diffusivity on PMI (MD, D_ax_ and D_rad_) is observed in all tracts except the PLIC. The bottom row of Fig. 9 shows this regression on the mean across the five white matter ROIs. The multiple regressions onto PMI and SI simultaneously indicate that PMI is the primary driving factor, and that correlations with MD, Dax and D_rad_ are significant (with multiple comparison correction, see Table 2). The diffusivity is reduced by 0.01–0.02 × 10^− 3^ mm^2^/s/h; however, extrapolating the regression curves to a PMI of zero hours does not yield common *in vivo* values, indicating either a non-linear dependence on PMI, or that additional factors (such as death itself) may contribute to these changes. FA only depends significantly on PMI for the SLF. The one white-matter region that is essentially independent of PMI is the posterior limb of the internal capsule, but this region does have a trend toward correlation with SI. While the PMI seems to have a greater effect on diffusion indices than SI, it may be that a true dependence on SI simply does not reach significance in our regression due to the low degrees of freedom (11 − 3 = 8) and correlation between PMI and SI. Some previous work does suggest that SI may not have a major effect on MD (Yong-Hing et al., 2005; Dyrby et al., 2011).

A further effect is the time it takes for fixative to diffuse, which is clearly not accounted for with a single (minimum) PMI, which does not account for the time it takes for fixative to permeate through the brain. This is likely another difference from smaller animal brains. Consistent differences between tracts (*e.g.*, finding highest FA in the CC) could in part reflect consistent differences in fixation times. For example, the PLIC is effectively the deepest structure studied with respect to distance to the surfaces in contact with fixative. If the fixative has to diffuse the longest distance to reach the PLIC, the effective PMI could be considerably longer than for other structures.

### Diffusion properties: other effects

The dependence of diffusion parameters on PMI suggests that some of the differences between *post mortem* and *in vivo* tissues are related to degradation, for example from autolysis in the period prior to fixation. It is clearly also possible that changes in diffusion characteristics could reflect changes in the size, geometry or exchange of restrictive spaces as a direct result of protein cross-linking during fixation (Shepherd et al., 2009). These effects are known to depend on the type and concentration of fixative used, and differences in diffusion properties measured with MRI have been convincingly demonstrated to depend primarily on fixation protocol (Shepherd et al., 2009). The cross-linking process can actually introduce structure by linking membrane proteins to intra- or extra-cellular proteins, and can disrupt membranes, leading to an increase in cross-membrane exchange (Shepherd et al., 2009). In addition to altering MD, increases in exchange across axon membranes could differentially increase diffusion across fibers compared to along fibers, leading to a reduction in FA.

Another important difference between our study and previous work on small-bore scanners is the requirement for long T_E_ in order to achieve significant diffusion weighting. One implication of this is that shorter T_ 2_ species will contribute less fractional signal compared to previous work, which could affect apparent diffusivity by selectively attenuating compartments with different diffusion properties. For example, the myelin sheath is associated with both very short T_ 2_ (MacKay et al., 2006) and anisotropic diffusion. Myelin is not the primary determinant of diffusion anisotropy and is unlikely to fully explain our results (Beaulieu, 2002); however, similar compartmental effects (*e.g.*, differences between intra- and extra-cellular compartments) could play a role.

Another complicating factor is the general dependence of apparent diffusion coefficient on b-value. The diffusion-weighted signal is well established to be non-mono-exponential (Niendorf et al., 1996), meaning that our mono-exponential analysis leads to an apparent dependence of ADC on b-value. Bi-exponential diffusion analysis typically fits a slower diffusion coefficient that is about 10 times lower than the fast diffusion coefficient, which is in good agreement with the discrepancy between our *post mortem* experiments and common *in vivo* measurements. However, at our b-value of 4500 s/mm^2^, the signal has only slightly departed from mono-exponential behavior (Niendorf et al., 1996; Mulkern et al., 1999; Sehy et al., 2002), and dominance of the slow diffusion component only happens at considerably higher b-value. Further, both fast and slow diffusion coefficients have been observed to be reduced in *post mortem* tissue (Niendorf et al., 1996). Given the factor of 10 reduction in MD that we measure, our b-value of 4500 s/mm^2^ is expected to have reduced diffusion contrast (fractional signal change) compared to standard *in vivo* protocols with b = 1000–1500 s/mm^2^ (Smith et al., 2007). The b-value used in our study was limited by hardware considerations on the clinical scanner.

A final possible source of alterations to diffusion indices is tissue pathology. The purpose of this study is to present methodology rather than study a particular disease or condition, and as such would ideally be limited to control brains with no known pathology (although the concept of a proper control is naturally problematic with human *post mortem* samples). However, due to the difficulties in obtaining control specimens, all of the brains presented in this study except one had a known pathology, primarily MS (eight of 11 brains). While care was taken in the ROI analysis above to avoid conspicuous lesions, the reduced MD and FA may in part reflect pathology. However, *in vivo* studies comparing normal-appearing white matter in MS have found reductions in FA of only 2–10% (Bammer et al., 2000; Ciccarelli et al., 2001), whereas FA in our brains is reduced by 50–60% compared to healthy, *in vivo* subjects (in white matter: 0.22–0.33 versus 0.6–0.8 (Snook et al., 2007)). Furthermore, a simple scatter plot comparing FA and MD of each subject and ROI does not suggest that the control brain (CTL01) differs in its diffusion properties from the brains with known pathology (see Supplementary Fig. 3).

### Observations in gray matter

**Fig. 10:**
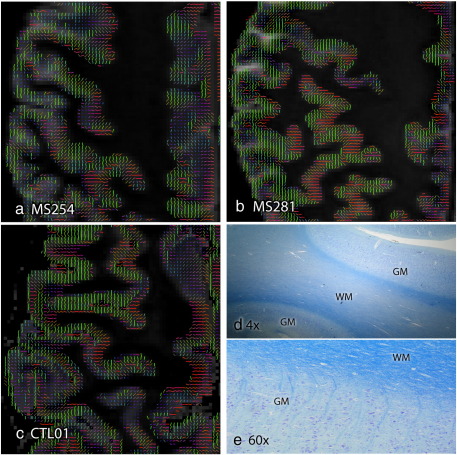
(a–c) The principal diffusion direction demonstrates a clear structure within the gray matter, running approximately perpendicular to the cortical surface in most regions. Here, the PDD is shown within a gray matter mask overlaid on the mean diffusivity map in three subjects. (d–e) These diffusion results can be compared to histological stains of human temporal lobe (different tissue specimen) using Cresyl violet and Luxol fast blue stains. An increase in fiber density is observed at the gray–white border. At higher magnification a more complicated fiber pattern is observed at the tissue boundary and radially oriented axons are visible in the cortex.

Although the above discussion is primarily focused on white matter, our data also show interesting effects in gray matter. Gray matter anisotropy is seldom visible in standard *in vivo* acquisitions (Shimony et al., 1999; Sorensen et al., 1999). However, our *post mortem* diffusion data displays coherent patterns of anisotropy in the cortex with high consistency. The principal diffusion direction in the cerebral cortex is oriented perpendicular to the pial surface in most regions (Fig. 10).

Radial diffusion has been reported at early stages in cortical development when fractional anisotropy is high (FA = 0.4–0.7) (Thornton et al., 1997; Mori et al., 2001; McKinstry et al., 2002; deIpolyi et al., 2005; Huang et al., 2006, 2008; Kroenke et al., 2007). This finding has been hypothesized to reflect diffusion restriction imposed by radial glia (which provide the scaffolding for neuronal migration) and pyramidal cells that have yet to form basal dendrites (Mori et al., 2001; McKinstry et al., 2002; Huang et al., 2006). As cortex matures, radial glia disappear and complicated patterns of dendritic branching are established, coincident with a reduction in FA. Our data and that of a few other studies (D'Arceuil et al., 2005; McNab et al., 2009a; Dyrby et al., 2011; Heidemann et al., 2010) suggest that some residual radial dominance persists, while other work has reported dominant diffusion parallel to the cortical surface (Golay et al., 2002). A preliminary report studying this phenomenon in greater detail suggests that both (radial and parallel) structure may exist, and may in fact be a microstructural marker that could be used to differentiate major brain regions (McNab et al., 2011). This structure could reflect diffusion restriction related to the mini-columnar structure of pyramidal cells, most of which retain their radial orientation through the cortical layers in maturity (see Fig. 10). However, it is unclear whether this particular cell population would represent sufficient structure to drive this signal behavior, or if other structural elements could be responsible.

**Fig. 11:**
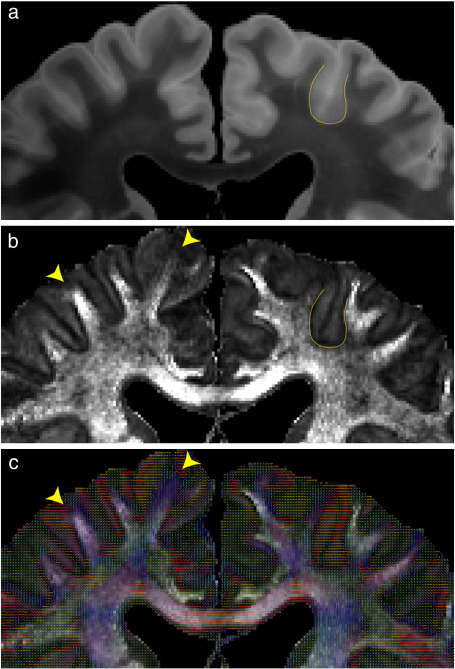
Demonstration of diffusion properties in cortex by comparing (a) structural, (b) FA and (c) PDD maps. FA exhibits a characteristic dark band at the interface between gray and white matter. The approximately matched distortion in the diffusion and structural data allows the cortical surface to be determined from the structural and overlaid on the FA map (yellow line). This comparison demonstrates that the dark band lies entirely in the gray matter, and varies from 0–1.4 mm (0–2 voxels) thick. The dark band is strongest where the PDD in adjacent gray and white matter are perpendicular (on the sulcal walls) and disappears at the ends of the gyri where the white matter tracts continue straight into the gray matter (arrowheads).

Another striking observation is a drop in FA near the gray–white border (Fig. 11). A superposition of the FA map on the aligned structural image indicates that this dark band lies within the gray matter, and thus likely represents the cortical layer closest to the boundary with white matter (Fig. 11). The alignment between structural and diffusion data is of high fidelity due to the low levels of distortion in the diffusion data, and that similar results can be obtained by superimposing the FA on the MD map. This dark band has been commented on in previous studies of fixed human brain tissue (D'Arceuil et al., 2005) and *in vivo* cat brain (Ronen et al., 2005), and can be discerned in several other studies (D'Arceuil et al., 2007; McNab et al., 2009a; Tyszka and Frank, 2009). However, the cause of this decreased FA is not well understood. Observation of this effect *in vivo* suggests that it is not simply caused by an alteration to the tissue during the process of fixation.

It has been hypothesized that the observed drop in FA could be caused by a sharp change in fiber direction as efferent fibers diverge from the main tract and enter gray matter perpendicular to the cortical surface (Ronen et al., 2005). This explanation is consistent with the observation of a prominent low FA band at sulcal walls, where tracts would turn sharply as they enter the cortex, and less evident band at gyral crowns, where tracts would continue straight into cortex. However, it would seem less consistent with the finding that the dark band is confined to gray matter, rather than existing at the boundary between gray and white matter (Fig. 11). Further, the band of decreased FA in our data is often several voxels thick (Fig. 11), indicating that fibers entering a given region of cortex would have to be drawn from across several millimeters of white matter.

An alternative possibility is that the low-FA band simply reflects the microstructural characteristics of the cortical layers bordering white matter (layers V and VI). Layer VI (the “multi-form” layer) is composed of a much broader range of cell types than the other cortical layers, which could reduce its microstructural coherence and lead to reduced FA (Briggs, 2010). This layer contains pyramidal cells oriented both parallel and perpendicular to the cortical surface, unlike other layers which almost exclusively contain perpendicular pyramidal cells (Briggs, 2010). The less prominent dark band at the gyral crowns could be related to the increased thickness of layer VI, resulting in elongated micro-columns with a more radial structure than is found in sulcal folds (Chance et al., 2004).

### Partial volume effects

Much of the potential worth of *post mortem* diffusion scanning lies in its ability to inform us about the properties of *in vivo* diffusion data, including interpretability of diffusion tensor indices and validation of tractography. In this final section, we describe one additional use of high-resolution diffusion data: to understand the relatively complicated interaction of fractional anisotropy and partial volume effects.

Fractional anisotropy is a commonly used measure of white matter integrity, despite having important limitations regarding its biological interpretation. While much work has focused on the range of microstructural changes that can lead to changes in FA (Beaulieu, 2002), this quantity also depends in an important way on partial volume, which occurs on a more mesoscopic scale. In this section, we simulate how partial volume effects (local averaging over the tissue within a voxel) can alter the apparent FA. The 0.73 mm DW-SE acquisition was analyzed to simulate how the tissue structures would appear at more common *in vivo* resolutions. The raw data was blurred with a Gaussian kernel to achieve an effective resolution of 2 × 2 × 2 mm and 3.5 × 3.5 × 3.5 mm, before re-fitting the diffusion tensor model to the blurred raw data. This analysis derives the FA that would result from data acquired at these lower resolutions. We visualize the resulting partial volume effects on the original 0.73 mm grid (rather than a 2 or 3.5 mm grid), as it is easier to interpret.

**Fig. 12:**
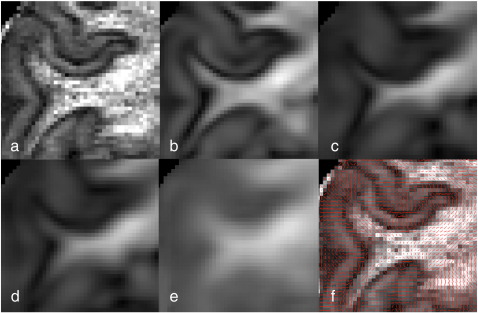
Partial volume effects at different image resolutions, as illustrated using high-resolution (0.73 mm isotropic) *ex vivo* diffusion data. (a) FA of original “ground truth” data. Simulated FA at lower resolution is obtained by blurring the raw data to recreate (b) 2 mm and (c) 3.5 mm resolution. These simulations indicate that at “normal” resolution, the tract appears thinner than it actually is due to partial-volume effects at the white–gray boundary. Note that the effects of blurring the individual elements of the high-resolution tensor matrix (d) or the FA map (e) to 2 mm resolution do not accurately predict these partial volume effects (with the blurred FA being particularly inaccurate). This is because the details of partial voluming depend critically on the underlying diffusion processes (f), and must encapsulate effects like cortical anisotropy and the low-FA band.

In Fig. 12a we see a cortical region-of-interest of the FA image derived from the original data, exhibiting high-FA white matter, medium-FA cortical gray matter, and the dark band at the gray–white boundary (the underlying directionality is also depicted in Fig. 12f). Figs. 12b and c simulate FA maps at the effective resolution of 2 and 3.5 mm. The white matter tracts appear thinner than they actually are (as judged by comparison with Fig. 12a) due to partial-volume averaging with voxels on the gray–white boundary. These voxels include gray matter containing anisotropy perpendicular to that in the white matter, thus strongly reducing the FA. Note that these partial volume effects cannot be accurately predicted by simply blurring the high-resolution FA map (Fig. 12e). Rather, the details of tract thinning and the appearance of the low-FA band depend crucially on the local diffusion directionality (although even blurring the individual tensor elements is not particularly accurate, see Fig. 12d).

These results illustrate that at typical resolutions obtained *in vivo*, tracts may appear thinner than they actually are due to partial volume effects. These results have implications for voxel-wise study of diffusion-based metrics, particularly voxel-based morphometry (VBM) or tract-based spatial statistics (TBSS) (Smith et al., 2006). Unless tracts are thick compared with voxel dimensions, it is not straightforward to tell whether apparent changes in FA are caused by changes in tract thickness or true underlying FA. Similarly, these results suggest that partial volume effects from adjacent gray matter can be fairly complicated due to the presence of cortical anisotropy, which will affect FA differently in the sulcal walls compared to gyral crowns, as shown above.

## Conclusions

We have demonstrated the feasibility of diffusion imaging of whole, *post mortem* human brains using a clinical 3 T scanner. In the present work, we used a fairly straightforward modification of the conventional DW-SE sequences that are commonly used *in vivo*. Studies at our institution currently use a 24-hour protocol centered around the 18-hour 3D DW-SE scan at 0.94 × 0.94 × 0.94 mm resolution. We have also demonstrated the ability to acquire data at 0.73 × 0.73 × 0.73 mm resolution, although this required a 5-day acquisition. Our data suggest that care must be taken in interpreting diffusion indices from *post mortem* human brains due to dependence of diffusion indices on *post mortem* and scan intervals. Nevertheless, our data is of sufficient quality to provide excellent visualization of white and gray matter anisotropy and to enable diffusion tractography. Our data also reveal intriguing radial diffusivity in the gray matter that could relate to important cortical microstructure.

## Figures and Tables

**Table 1:** Mean value for tensor-derived parameters across eleven *post mortem* brains from four white matter and three gray matter regions. Diffusivities are given in mm^2^/s × 10^− 3^. Subject-wise values are available in the Supplementary Material.

	CC	PLIC	SLF	Opt	Cing	Thal	Caud	Put
FA	0.32 (0.08)	0.22 (0.03)	0.25 (0.05)	0.26 (0.03)	0.28 (0.04)	0.12 (0.02)	0.09 (0.02)	0.10 (0.02)
MD	0.074 (0.023)	0.084 (0.022)	0.094 (0.021)	0.076 (0.018)	0.072 (0.017)	0.150 (0.021)	0.184 (0.035)	0.175 (0.031)
D_ax_	0.098 (0.028)	0.102 (0.025)	0.116 (0.026)	0.097 (0.024)	0.092 (0.021)	0.166 (0.023)	2.01 (0.039)	0.191 (0.034)
D_rad_	0.062 (0.022)	0.075 (0.021)	0.082 (0.020)	0.065 (0.016)	0.061 (0.014)	0.141 (0.020)	0.175 (0.034)	0.167 (0.030)

**Table 2:** Effect of PMI and SI on diffusion indices. Reported regression coefficients reflect change in diffusion parameter per hour PMI or per month SI (diffusivity is in 10^− 3^ mm^2^/s). Stars indicate significance (*p ≤ 0.05, corrected). Regressions coefficients with p > 0.25 (corrected) are not reported.

		CC	SLF	Opt	Cing	PLIC	Average
PMI	FA		− 0.019 *				
MD	0.0146 *	0.0172 *		0.0145 *		0.0122 *
D_ax_	0.0180 *	0.0193 *		0.0186 *		0.0149 *
D_rad_	0.0130	0.0164 *		0.0126 *		0.0111 **
							

SI	FA		0.022 *			0.028	
MD	0.0094	− 0.0115			− 0.0091	
D_ax_	0.0104	− 0.0118				
D_rad_	0.0119	− 0.0132			− 0.0123	
