# Supplementary material for: Diffusion imaging of whole, post-mortem human brains on a clinical MRI scanner
Source: Neuroimage. 2011 Jul 1;57(1-4):167–81. doi: 10.1016/j.neuroimage.2011.03.070 (PMC3115068; doi:10.1016/j.neuroimage.2011.03.070)
Supplement: Supplementary file 1 — Supplementary Material. [file mmc1.pdf]

subject	CC	PLIC	SLF	Opt	Cing	Thal	Caud	Put
ASD02	0.353 (0.154)	0.209 (0.052)	0.263 (0.062)	0.267 (0.059)	0.291 (0.057)	0.108 (0.032)	0.062 (0.020)	0.056 (0.017)
CTL01	0.427 (0.098)	0.212 (0.047)	0.313 (0.077)	0.281 (0.056)	0.232 (0.041)	0.095 (0.034)	0.112 (0.022)	0.106 (0.030)
MND01	0.254 (0.069)	0.181 (0.040)	0.246 (0.053)	0.248 (0.058)	0.201 (0.058)	0.107 (0.027)	0.084 (0.029)	0.082 (0.025)
MS254	0.353 (0.182)	0.227 (0.061)	0.228 (0.059)	0.304 (0.065)	0.330 (0.083)	0.120 (0.032)	0.074 (0.020)	0.081 (0.026)
MS281	0.453 (0.122)	0.194 (0.039)	0.249 (0.062)	0.308 (0.091)	0.295 (0.040)	0.121 (0.028)	0.123 (0.030)	0.090 (0.025)
MS314	0.309 (0.085)	0.171 (0.048)	0.230 (0.086)	0.245 (0.053)	0.265 (0.071)	0.113 (0.023)	0.121 (0.025)	0.098 (0.025)
MS316	0.280 (0.086)	0.240 (0.060)	0.188 (0.086)	0.260 (0.075)	0.283 (0.077)	0.117 (0.042)	0.072 (0.024)	0.097 (0.027)
MS322	0.248 (0.076)	0.223 (0.083)	0.246 (0.108)	0.253 (0.070)	0.278 (0.090)	0.115 (0.035)	0.082 (0.027)	0.107 (0.031)
MS332	0.205 (0.060)	0.219 (0.069)	0.172 (0.065)	0.247 (0.079)	0.248 (0.084)	0.114 (0.037)	0.097 (0.032)	0.101 (0.029)
MS334	0.268 (0.100)	0.288 (0.093)	0.326 (0.129)	0.245 (0.086)	0.317 (0.098)	0.161 (0.038)	0.121 (0.037)	0.123 (0.043)
MS400	0.323 (0.124)	0.251 (0.093)	0.234 (0.082)	0.212 (0.068)	0.326 (0.102)	0.148 (0.052)	0.090 (0.032)	0.104 (0.033)
all	0.316 (0.076)	0.220 (0.033)	0.245 (0.045)	0.261 (0.028)	0.279 (0.040)	0.120 (0.019)	0.094 (0.022)	0.095 (0.018)

TABLE 1: Supplementary material. Fractional anisotropy for eleven *post mortem* brains from four white matter and three gray matter regions.

subject	CC	PLIC	SLF	Opt	Cing	Thal	Caud	Put
ASD02	0.0534 (0.0170)	0.0911 (0.0049)	0.0893 (0.0110)	0.0839 (0.0075)	0.0725 (0.0045)	0.1153 (0.0106)	0.1012 (0.0096)	0.1557 (0.0135)
CTL01	0.0354 (0.0069)	0.0915 (0.0145)	0.0851 (0.0160)	0.0480 (0.0086)	0.1033 (0.0093)	0.1758 (0.0149)	0.1969 (0.0108)	0.2093 (0.0196)
MND01	0.0572 (0.0151)	0.0686 (0.0074)	0.0682 (0.0120)	0.0553 (0.0065)	0.0644 (0.0066)	0.1579 (0.0141)	0.1733 (0.0158)	0.1751 (0.0144)
MS254	0.0743 (0.0272)	0.0882 (0.0087)	0.1035 (0.0207)	0.0896 (0.0167)	0.0728 (0.0087)	0.1746 (0.0300)	0.2147 (0.0157)	0.2143 (0.0243)
MS281	0.0649 (0.0177)	0.1086 (0.0055)	0.1035 (0.0148)	0.0849 (0.0112)	0.0914 (0.0066)	0.1477 (0.0146)	0.1788 (0.0145)	0.1892 (0.0167)
MS314	0.1086 (0.0266)	0.1304 (0.0170)	0.1423 (0.0289)	0.1068 (0.0190)	0.0841 (0.0097)	0.1804 (0.0237)	0.2195 (0.0322)	0.2147 (0.0210)
MS316	0.0877 (0.0195)	0.0511 (0.0083)	0.1059 (0.0833)	0.0594 (0.0158)	0.0429 (0.0049)	0.1219 (0.0390)	0.2236 (0.0329)	0.1397 (0.0148)
MS322	0.0852 (0.0162)	0.0814 (0.0210)	0.1047 (0.0377)	0.0836 (0.0216)	0.0672 (0.0112)	0.1461 (0.0185)	0.1914 (0.0143)	0.1627 (0.0160)
MS332	0.1111 (0.0406)	0.0807 (0.0192)	0.0739 (0.0130)	0.0821 (0.0296)	0.0731 (0.0183)	0.1461 (0.0407)	0.1863 (0.0493)	0.1833 (0.0172)
MS334	0.0730 (0.0200)	0.0592 (0.0112)	0.0720 (0.0272)	0.0548 (0.0216)	0.0613 (0.0107)	0.1392 (0.0213)	0.1480 (0.0173)	0.1166 (0.0143)
MS400	0.0593 (0.0161)	0.0712 (0.0187)	0.0811 (0.0196)	0.0827 (0.0155)	0.0557 (0.0109)	0.1398 (0.0333)	0.1900 (0.0093)	0.1663 (0.0164)
all	0.0736 (0.0232)	0.0838 (0.0223)	0.0936 (0.0214)	0.0755 (0.0183)	0.0717 (0.0168)	0.1495 (0.0212)	0.1840 (0.0351)	0.1752 (0.0313)

TABLE 2: Supplementary material. Mean diffusivity (in 10^{-3} mm^2/s) for eleven *post mortem* brains from four white matter and three gray matter regions.

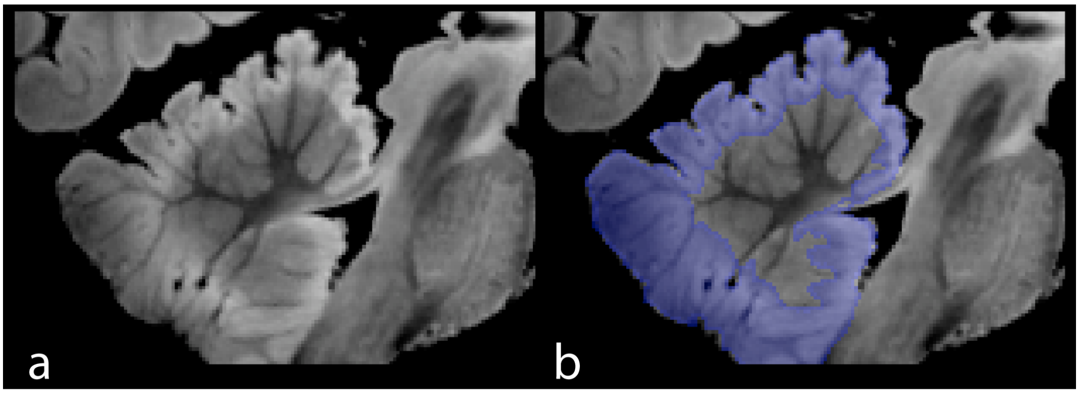

FIG. 1: Supplementary Material. (a) Structural scan of a *post mortem* brain that was soaked in a buffer solution for 24 hours prior to imaging. Soaking aims to increase tissue T_2 and thereby signal, which has been found to be beneficial on smaller, animal brains. However, the large size of human brains prevents fluid from fully penetrating in the allotted time. The resulting diffusion boundary creates contrast that confounds the visualization of tissue structure (e.g. the grey-white boundary). (b) This problem can be visualized more clearly by simple thresholding of the signal in the cerebellum, represented by the color overlay shown here. The appearance of the contrast boundary is similar to the fixation boundary reported previously [?].

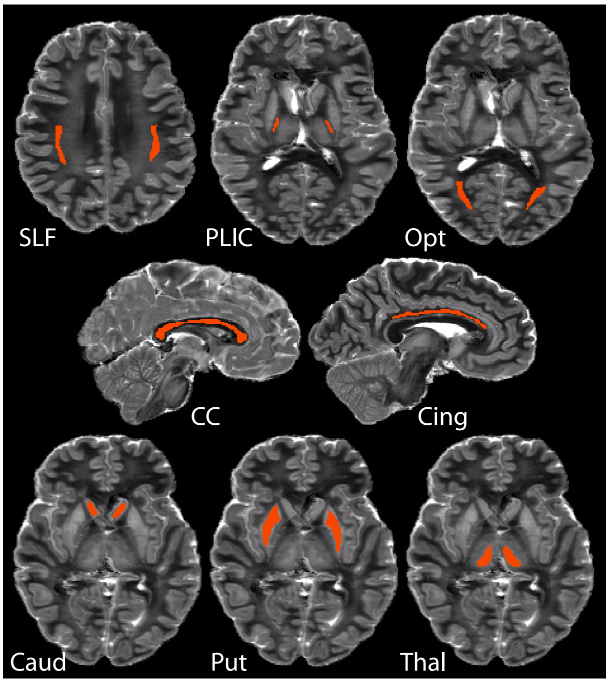

FIG. 2: Supplementary Material. Masks used for region-of-interest analyses (shown for subject CTL01).

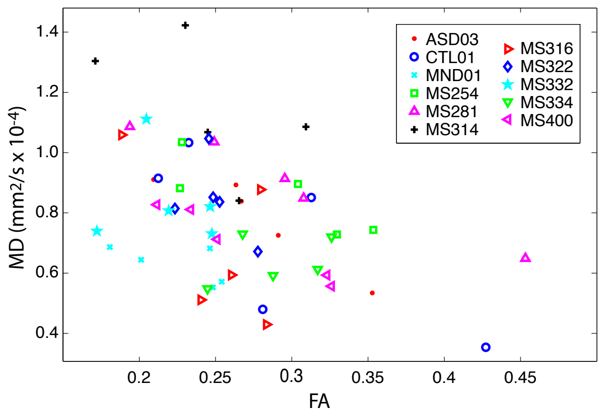

FIG. 3: Supplementary Material. Scatter plot of FA vs MD for each subject (indicated by symbols) and each ROI. There is no evidence of a relationship between pathology (or lack of known pathology in CTL01) and diffusion properties. There does appear to be an inverse relationship between MD and FA in many of the brains.

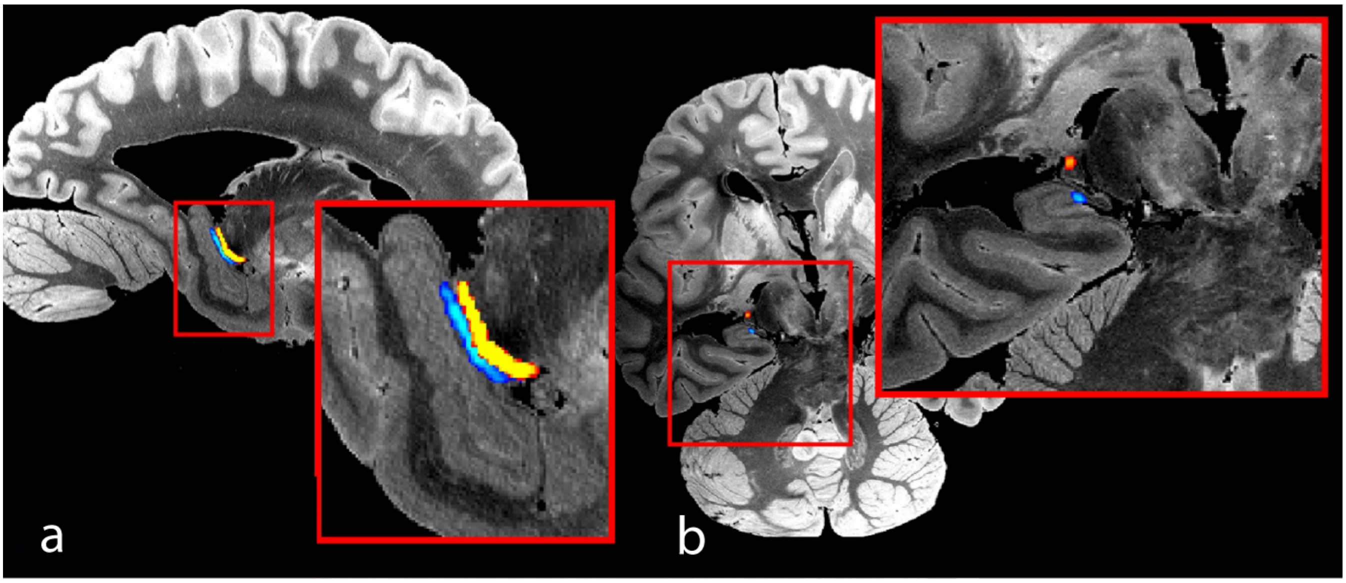

FIG. 4: Supplementary Material. Probablistic tractography of the inferior portion of the stria terminalis and fornix. Seed points were selected in the axial orientation, at the level of the lateral geniculate, with the stria terminalis seeded anterior to the posterior horn of the lateral ventricle and the fornix seeded posterior to the posterior horn of the lateral ventricle. Tractography results are superimposed on 330 μm isotropic structural images.
